# Emerging Xene-Related Nanostructures for Versatile Applications

**DOI:** 10.3390/nano13030517

**Published:** 2023-01-28

**Authors:** Mengke Wang, Weichun Huang

**Affiliations:** School of Chemistry and Chemical Engineering, Nantong University, Nantong 226019, China

Investigations into semiconductor nanomaterials from both an academic and industrial point of view are of great significance [1,2,3,4]. Xenes such as MXenes [4,5,6,7,8,9], phosphorene [10,11], graphdiyne (GDY) [12], tellurene [13], antimonene [14], and bismuthene [2,15], are important semiconductors that offer extraordinary properties, including high photoconductivity, anisotropic thermal conductivity, excellent photothermal efficiency, and high piezoelectric and thermoelectric response. Research on Xenes and Xene-related functional nanostructures in (opto)electronics, energy storage, sensors, catalysis, laser photonics, etc., are currently fascinating yet challenging research topics, which are expected to rapidly promote the development of new designs of high-performance devices.

This Special Issue compiles twelve articles dedicated to Xene-related nanostructures for versatile applications: ten research articles and two review articles. The papers present a variety of Xene-related nanostructures including two-dimensional (2D) black phosphorus (BP) nanosheets (NSs) [16,17], zero-dimensional (0D) bismuth (Bi) quantum dots (QDs) [18], one-dimensional (1D) telluride (Te) nanotubes (NTs) [19], 2D MXene [20,21,22], 0D graphdiyne (GDY) QDs [23], 2D selenium (Se) NSs [24], and other related nanostructures (2D WSe_2_ NSs [25], CH_3_NH_3_PbBr_3_ perovskite [26], and SnO_2_ nanoparticles [27]) are also featured. These advanced nanostructures have been widely applied to versatile applications, such as lithium (Li)-Te batteries [19], solar cells [20], electromagnetic interference (EMI) shielding [21], hydrophobic and photothermal surfaces [24], dental materials [18], optical devices [16,23,25], sensors [22,26], etc. The purpose of this Special Issue is to summarize the most recent advances in Xene-related nanostructures for a variety of edge-cutting applications. It targets a broad readership of materials scientists, chemists, dentists, environmentalists, and nanotechnologists. In the following paragraphs, we, as the guest editors of this Special Issue, provide a brief overview of the individual articles published and hope to inspire the interest of potential readers.

We open the discussion on the published review article that focuses on MXene-based solar cells by Shi et al. [20] This work aims to study those MXenes employed in solar technologies, including fabrication methods, electrical, optical, and thermoelectric properties, and solar cell application. Xia and Xiao reported MXene Ti_3_C_2_ with a loosely packed accordion-like structure, was fabricated and adsorbed by three metal ions (Fe^3+^/Co^2+^/Ni^2+^) [21]. Due to fact that the adsorbed metal ion can improve the absorption efficiency via electromagnetic wave scattering, the MXene/metal ion can largely improve the EMI shielding performance, which can hold great potential in communications, electronics, military, etc. A highly sensitive photothermal fiber sensor based on MXene Ti_3_C_2_T*_x_* NSs reported by Wu et al. [22], was successfully fabricated by directly depositing Ti_3_C_2_T*_x_* NSs onto the ring of a microfiber knot resonator (MKR) via an optical deposition method. The experimental results demonstrated the modulation efficiency of the MXene MKR and the modulation efficiency of the cascade system was 0.02 nm mW^−1^ and 0.15 nm mW^−1^, respectively. It was also verified that the sensitivity of the all-fiber photothermal sensor has many advantages such as low cost, small size, good system compatibility, and excellent sensitivity.

In recent years, novel Xene monoelemental nanostructures have exhibited unprecedented potential owing to their unique structures, and excellent physicochemical, optical, and electronic properties. The report by Wu et al. [23] applied 0D GDY QDs into modified tapered fibers for an erbium-doped fiber laser to achieve a femtosecond pulse output. The 0D GDY QD-based all-fiber saturable absorber device exhibited strongly saturable absorption characteristics with a modulation depth of 18.06% and a saturation intensity of 103.5 W. The net dispersion of the erbium-doped fiber laser cavity was ~0.016 ps^2^, and a femtosecond pulse output was achieved with a bandwidth of 26.3 nm, a pulse width of 135.8 fs, and a single pulse capability of 54 pJ. Uniform 1D Te NTs prepared through a facile hydrothermal method, were directly mixed with a small amount of nano fibrillated celluloses without extra conductive carbon or a binder to obtain a flexible and freestanding electrode for Li-Te batteries [19]. The as-prepared Te-based electrode exhibited a high volumetric capacity of 1512 mAh cm^−3^ at 200 mA g^−1^, and delivered superior cyclic stability with a capacity retention of 104% over 300 cycles and excellent rate performance of 833 mAh cm^−3^ at 1000 mA g^−1^. Surprisingly, even after being bent 50 times, the Te-based electrode could still deliver a desirable volumetric capacity of 1117 mAh cm^−3^, and remained at 93% of initial capacity after 100 cycles, suggesting that the as-fabricated Te-based electrode can serve as a potential candidate for flexible Li-Te batteries. Moreover, Hu et al. [18] reported that 0D Bi QDs were employed to fabricate a Bi QD/polydimethylsiloxane (PDMS)-modified tooth by simple curing treatment to achieve self-cleaning and antibacterial activity for dental applications. The as-fabricated BiQD/PDMS-modified tooth at 37 °C for 120 min not only showed significantly improved hydrophobic performance with remarkably enhanced water contact angles (103° on the tooth root and 115° on the tooth crown), respectively, compared to those (~20° on the tooth root, and ~5° on the tooth crown) of the pristine tooth, respectively, but also exhibited excellent antibacterial activity, superior biocompatibility, and biosafety. Owing to the highly photothermal effect of Bi QDs, the antibacterial activity of the as-fabricated Bi QD/PDMS-modified tooth could be further enhanced under illumination, even at a very low power density (12 mW cm^−2^). In addition, 2D Se NSs, reported by Chen et al. [24], is a contribution from the guest editor. They successfully fabricated a composite (Se@MS) consisting of 2D Se NSs and commercially used melamine sponge (MS) by a facile dip-coating method. The as-obtained Se@MS exhibited a rapid wettability transition from hydrophilicity to hydrophobicity, and the highly stable photothermal conversion with a maximum temperature of 111 ± 3.2 °C due to the excellent photothermal effect of 2D Se NSs, which affords new design strategies for multifunctional porous structures for versatile applications, such as high-performance photothermal sterilization and solar desalination. Xene monoelemental nanostructure-based functional nanostructures were also rationally designed and successfully fabricated to further boost the performance, such as BP/InSe heterostructure [16]. The report by Shu et al. [16] utilized high hole mobility of BP and high electron mobility of InSe, to fabricate BP/InSe heterostructure by a liquid-phase exfoliation method, which was embedded into an erbium-doped fiber laser. The harmonic mode-locking of bound solitons and dark-bright soliton pairs was obtained in the same laser cavity due to the cross-coupling effect, and the stable mode-locked operation could be well kept for several days, greatly improving the environmental stability of BP in air.

Other Xene analogues, such as WSe_2_ [25], SnO_2_ [27], and CH_3_NH_3_PbBr_3_ perovskite [26], also show intriguing performance in optic devices. The report by Chen et al. [25] focused on all-optical devices based on 2D WSe_2_ NSs, combined with drawn tapered fibers as saturable absorbers to achieve ultrashort pulse output. The all-optical 2D WSe_2_ NS-based device displayed excellent saturable absorption characteristics with a modulation depth of 15% and a saturation intensity of 100.58 W. The net dispersion of the erbium-doped fiber laser cavity was ~−0.1 ps^2^, and a femtosecond pulse output had a bandwidth of 11.4 nm, a pulse width of 390 fs, and a single-pulse capability of 42 pJ, which demonstrated that 2D WSe_2_ NSs as saturable absorbers could be an effective photonic device for realizing stable fiber lasers. Size-selected SnO_2_ NPs fabricated by a liquid cascade centrifugation technique reported by Huang et al. [27], were, for the first time, directly deposited onto a poly(ethylene terephthalate) film with a regular Ag lattice to prepare a flexible working electrode for a photoelectrochemical (PEC)-type photodetector. The PEC result verified that the SnO_2_ NP-based PEC electrode presented a superior photocurrent density (14.0 µA cm^−2^), and excellent self-powered photoresponse performance as well as stable photodetection under ambient conditions, which offered diverse availabilities for high-performance self-powered optoelectronic devices to realize a carbon-neutral or carbon-free environment. Wu et al. [26] used high-refractive-index dielectric films (CH_3_NH_3_PbBr_3_ perovskite) as the guided-wave (GW) layer, which combined with the long-range surface plasmon resonance (LRSPR) structure to form the GW-LRSPR sensing structure. The theoretical results demonstrated that the LRSPR signal was enhanced by the additional high-RI GW layer, and the experimental results exhibited the highest sensitivity (*S* = 1340.4 RIU^−1^), and the corresponding figure of merit (8.16 × 10^4^ RIU^−1^ deg^−1^). Note that the sensitivity of this new sensor was enhanced by ~94% compared with the conventional LRSPR sensor (*S* = 688.9 RIU^−1^). The last paper reported by Li et al. [17] reviewed the recent progress of 2D materials in infrared light emission device applications, including background and motivation, 2D-material-based spontaneous emission and laser application, and challenges and future opportunities.
